# Dairy products consumption and metabolic syndrome in adults: systematic review and meta-analysis of observational studies

**DOI:** 10.1038/srep14606

**Published:** 2015-09-29

**Authors:** Guo-Chong Chen, Ignatius M. Y. Szeto, Li-Hua Chen, Shu-Fen Han, Yan-Jie Li, Rina van Hekezen, Li-Qiang Qin

**Affiliations:** 1 Department of Nutrition and Food Hygiene, School of Public Health, Soochow University, Suzhou, 215123, China; 2 Yili Innovation Center, Inner Mongolia Yili Industrial Group Co., Ltd., Hohhot, 010110, China; 3 Yili R&D Center (The Netherlands) B.V., 6708 PB Wageningen, Netherlands

## Abstract

The association of dairy products consumption with risk of metabolic syndrome (MetS) has been inconsistently reported in observational studies. A systematic review and meta-analysis of published observational studies was conducted to quantitatively evaluate this association. Relevant studies were identified by searching PubMed and EMBASE databases and by carefully checking the bibliographies of retrieved full reports and related reviews. Eligible studies were observational studies that investigated the association between dairy products consumption and risk of MetS in adults, with risk estimates available. Random-effects model was assigned to calculate the summary risk estimates. The final analysis included 15 cross-sectional studies, one case-control study and seven prospective cohort studies. Higher dairy consumption significantly reduced MetS by 17% in the cross-sectional/case-control studies (odds ratio = 0.83, 95% confidence interval [CI], 0.73–0.94), and by 14% (relative risk [RR] = 0.86, 95% CI, 0.79–0.92) in cohort studies. The inverse dairy-MetS association was consistent in subgroup and sensitivity analyses. The dose-response analysis of the cohort studies conferred a significant 6% (RR = 0.94, 95% CI, 0.90–0.98) reduction in the risk of MetS for each increment in dairy consumption of one serving/d. No significant publication bias was observed. Our findings suggest an inverse dose-response relationship between dairy consumption and risk of MetS.

Metabolic syndrome (MetS) consists of a cluster of cardiovascular risk factors including central obesity, high blood pressure (BP), hyperglycemia, hypertriglyceridemia, and low high-density lipoprotein (HDL) cholesterol levels^1^. MetS is found to be associated with increased risks of cardiovascular disease (CVD), type 2 diabetes mellitus (T2DM) and all-cause mortality, as well as specific cancers^2^,^3^,^4^. Recently, the prevalence and incidence of MetS have rapidly increased worldwide, and the increase has mostly been attributed to the influence of Western lifestyle characterized by high consumption of red and processed meat, refined grains and fried foods, and low physical activity^5^. Dietary and lifestyle factors clearly play a role, but to what extent these factors influence the development of MetS has continuously been a question open for investigations.

With respect to dietary factors, dairy products have long been suspected to prevent the individual components of MetS^6^,^7^,^8^,^9^. However, the relationship between dairy products consumption and overall risk of MetS remains unclear. According to the 2010 Dietary Guidelines for Americans^10^, there is “moderate evidence” showing that dairy products is beneficial for CVD, T2DM and BP in adults, but the role of dairy in protecting against MetS needs to be determined in further researches. A recent review^11^ (without meta-analysis) summarized observational studies published up to 2009 and indicated that the evidence for the benefits of dairy consumption on MetS was suggestive, but limited by methodological problems of primary studies. However, the nature and the extent of the relationship between dairy consumption and risk of MetS remain unknown, and many other studies were missed or not available at that time. Given the common consumption of dairy products and high presence of MetS throughout the world, it is relevant for public health to elucidate how dairy consumption affects MetS development. In an attempt to clarify this issue, a comprehensive systematic review and meta-analysis summarizing published observational studies was carried out.

## Results

### Study selection

**Figure 1:**
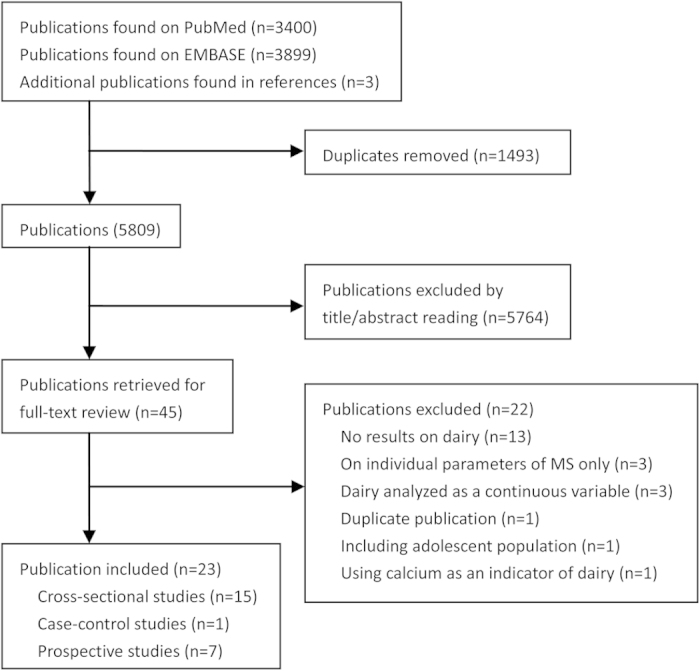
Flow chart of study selection.

A flow chart of study screening and selection process is reported in Fig. 1. Briefly, a total of 5809 independent citations were identified after duplicates exclusion, of which 45 were retrieved for more detailed reviews. Twenty two reports were excluded after carefully reading the full texts (Supplementary Table S1). Finally, a total of 23 publications, including 15 cross-sectional studies^12^,^13^,^14^,^15^,^16^,^17^,^18^,^19^,^20^,^21^,^22^,^23^,^24^,^25^,^26^, one case-control study^27^ and seven prospective cohort studies^28^,^29^,^30^,^31^,^32^,^33^,^34^ were included in this meta-analysis.

### Study characteristics

The characteristics of the cross-sectional/case-control studies are summarized in Supplementary Table S2. The 16 studies were published between 2000 and 2014. They were from the USA (*N* = 3), Mexico (*N* = 1), Brazil (*N* = 1), the UK (*N* = 2), France (*N* = 2), the Netherlands (*N* = 1), Iran (*N* = 3), Korea (*N* = 2) and China (*N* = 1). Three studies included men only, two studies included women only, and 11 studies consisted of both sexes. Twelve studies assessed total dairy products, three studies examined milk, and one study included cheese only. Fourteen studies were published in English, one in Chinese and one in Persian. All but one study were published in full reports. The characteristics of the cohort studies are reported in Supplementary Table S3. The seven studies were published between 2002 and 2013. Two of them were from the USA, and the remaining were five cohorts each from France, the Netherlands, Australia, Japan and Korea. All studies recruited both men and women, and all but one cohort examined total dairy consumption (or total dairy except cheese in one). Most included studies applied self-administered food frequency questionnaires (FFQ) to estimate dairy consumption, and diagnosed MetS according to the NCEP ATP-III criteria. All studies reported multivariable-adjusted risk estimates.

### Meta-analysis of cross-sectional/case-control studies

**Figure 2:**
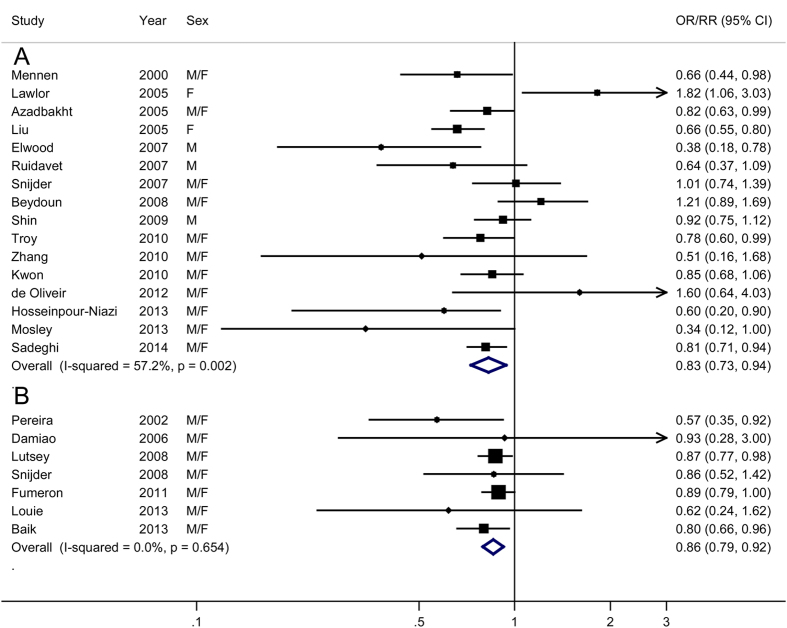
Meta-analysis of dairy products consumption (high vs. low) and risk of metabolic syndrome. (**A**) cross- sectional and case-control studies; (**B**) prospective cohort studies; M, male; F, female. OR, odds ration; CI, confidence interval.

A meta-analysis of the 16 cross-sectional/case-control studies suggested that subjects in the highest categories of dairy consumption had a 17% reduction in the risk of MetS (OR = 0.83, 95% CI, 0.73–0.94), with moderate heterogeneity among studies (*P* = 0.002, *I*^2^ = 57.2%) (Fig. 2A). There was no evidence of publication bias (*P* for Egger’s test = 0.84).

### Meta-analysis of prospective cohort studies

A meta-analysis of the seven prospective cohort studies yielded a summary RR of 0.86 (95% CI, 0.79–0.92) (Fig. 2B), with no evidence for heterogeneity (*P* = 0.65, *I*^2^ = 0.0%) or publication bias (*P* for Egger’s test = 0.13).

### Subgroup and sensitivity analyses

In general, the inverse association between dairy consumption and risk of MetS was consistently observed in the subgroup analysis stratified by pre-defined factors (Table 1). For cross-sectional/case- control group, excluding the only case-control study (OR = 0.83, 95% CI, 0.73–0.95), restricting to full reports (OR = 0.83, 95% CI, 0.72–0.96), or to English-published full reports (OR = 0.84, 95% CI 0.73–0.98) did not materially change our primary results. A sensitivity analysis in which studies were omitted one at a time with the remaining studies pooled further confirmed the robustness of our findings (Supplementary Figure S1 and 2). In a further analysis including all independent studies (*N* = 21, excluding two overlapping cross-sectional studies^28^,^31^), the summary OR was 0.83 (95% CI, 0.76–0.90), with moderate heterogeneity (*P* = 0.01, *I*^2^ = 46.1%), but no publication bias (*P* for Egger’s test = 0.40). No additional meta-analyses for individual dairy products rather than milk could be performed because the reported results were too limited and heterogeneous (Supplementary Table S4).

### Dose-response analysis

**Figure 3:**
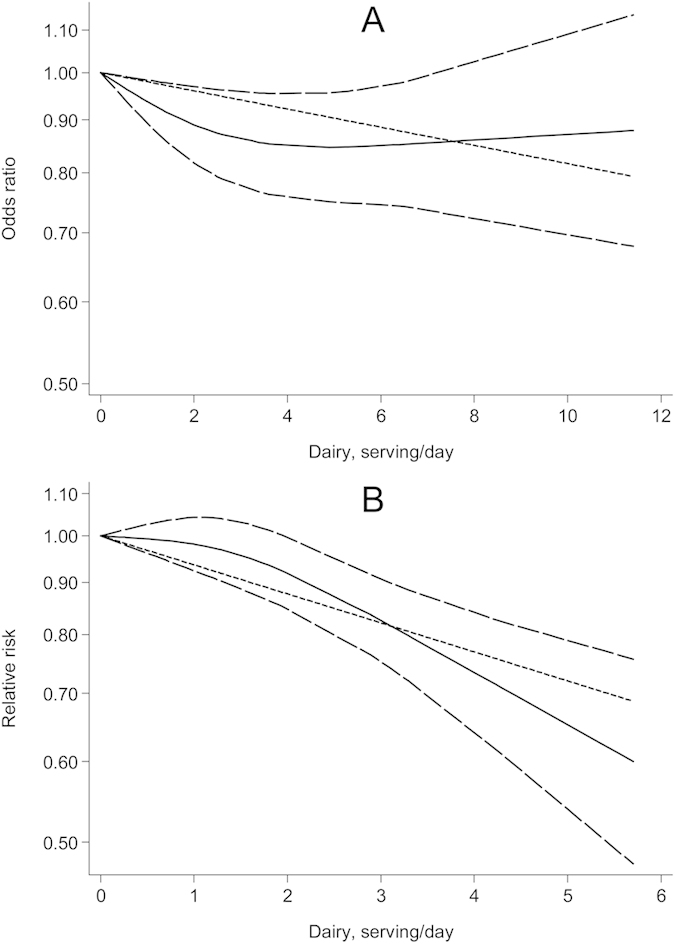
Risk estimates with 95% confidence interval (long dashed lines) for the association between dairy products consumption and risk of metabolic syndrome in a restricted cubic spline random-effects meta-analysis. (**A**) cross-sectional and case-control studies; (**B**) prospective cohort studies.

In cross-sectional/case-control studies (*N* = 8^12^,^13^,^16^,^19^,^23^,^24^,^26^,^27^), dairy consumption was associated with reduced risk of MetS (OR per one serving/d = 0.98, 95% CI, 0.94–1.01) in a somewhat U-shaped fashion (*P* for linearity = 0.045, Fig. 3A), with the greatest risk reduction (~15%) observed at consumption of ~five servings/d. In prospective cohort studies (*N* = 5^28^,^29^,^31^,^32^,^33^), dairy consumption was linearly *(P* for linearity = 0.074, Fig. 3B) and inversely associated with MetS risk (RR per one serving/d = 0.94, 95% CI, 0.90–0.98), but the reductions in risk appeared more evidence when the consumptions were up to two servings/d. Excluding one cross-sectional study and one cohort study that reported results in weights had little impacts on these findings, with a summary OR and RR of 0.98 (95% CI, 0.94–1.01) and 0.94 (0.89–0.98), respectively.

## Discussion

Our study consistently showed a significant inverse association between dairy products consumption and risk of MetS. Higher dairy consumption was significantly associated with 17% and 14% reduced risk of MetS in cross- sectional/case-control studies and prospective cohort studies, respectively. The observed reductions persisted in the stratifications by multiple study characteristics including adjustment for potential confounders, suggesting that the inverse dairy-MetS relationship is probably an independent one. Furthermore, the dose-response analysis of prospective cohort studies estimated that each increase in dairy consumption of one serving/d significantly reduced MetS by 6%.

The dairy-MetS association has been inconsistently reported in individual studies. In cross-sectional/case-control studies, the association was inverse in 12 studies (eight of which reached statistical significance), positive in three (one was significant), and null in one. These between-study discrepancies are not surprising given the considerable differences in dairy categories, study populations and methodologies across the studies. To explore potential sources of heterogeneity, various stratified analyses were performed, and the results generally supported our overall findings. The evidence from prospective cohort studies appeared more consistent, with all seven studies reporting a risk estimate of less than unit, among which four was statistically significant. The prospective studies may provide more accurate estimates than retrospective studies because: first, they generally had a better control for potential confounders including socioeconomic, lifestyle and dietary factors that may both be related to dairy consumption and MetS development; second, they had a sufficient duration of follow-up to detect MetS cases (≥6 years); and further, they collected exposure information before the occurrence of diseases and, therefore, had ability to examine the temporal causal-relationship.

As a meta-analysis of published literature, publication bias that results from a tendency to publish only positive results is always a consideration. We used a broad search strategy, and carefully reviewed those publications on food groups/dietary factors and MetS, and considered those reports published as abstracts and those in non-English language. Furthermore, three excluded studies, in which dairy products were analyzed as a continuous variable, also supported the benefits of dairy on MetS (Supplementary Table S1). In addition, no significant publication bias was detected by the statistical test. Taken together, the potential influence of publication bias on our findings should be minimized.

Our findings are in line with previous evidence that certain dietary patterns including increases in dairy products consumption were beneficial for MetS^35^. The same is valid for dairy consumption being protective for diseases that could be a consequence of MetS, like T2DM^36^, stroke^37^, and cancers of breast^38^ and colorectum^39^. A recent meta-analysis^9^ of nine prospective studies showed a linear inverse relationship between dairy consumption and incidence of hypertension. The majority observational studies reported a protective impacts of dairy products on obesity^40^.

Recent meta-analyses^8^,^41^ of published randomized controlled trials (RCTs) found that high-dairy calorie-restricted diets lead to a significant loss in weight and body fat, and a reduction in waist circumference (WC), but dairy consumption without energy restriction did not, when comparing with conventional calorie-restricted diets, leading the investigators to hypothesize that the appetite-lowering effects of dairy may be more efficient together with energy-restricted diets. Total energy intake could increase due to increased dairy consumption^42^, and studies without a consideration of energy intake may fail to show an inverse dairy-MetS association. In the subgroup analysis, there were indications that the association between dairy consumption and risk of MetS was stronger in cross-sectional/case-control (OR: 0.78 *vs*. 0.99) and cohort studies (RR: 0.83 *vs*. 0.89) with adjustment for dietary energy intake, although the differences between strata were not significant (*P* ≥ 0.22).

Various constituents in dairy may contribute to the beneficial effects of dairy products on MetS, and the most extensively studied are calcium and vitamin D. Intracellular calcium can regulate lipid metabolism by its direct effects on adipocytes and thereby contribute to weight loss^43^. A recent meta-analysis of seven RCTs showed that calcium supplementation resulted in a significant reduction in body weight^44^. There is evidence that calcium intake has favorable impacts on the reductions of BP^45^, fasting glucose^24^, WC^45^, and triglyceride levels^46^, and the increase of HDL cholesterol levels^46^. Higher calcium intake has also been reported to be associated with lower presence or incidence of MetS in observational studies 18,30,45. Animal studies showed that vitamin D improved impaired glucose tolerance and insulin secretion^47^, and low concentrations can inhibit pancreatic secretion of insulin^48^. Several studies found that circulating concentration of 25-(OH) vitamin D, a good indicator of vitamin D status in human body, was positively associated with insulin sensitivity^49^, and inversely associated with the risk of MetS^50^. Other nutrients in dairy that may contribute to the prevention of MetS include magnesium, potassium and whey protein. Dietary magnesium^51^ and potassium^52^ have been reported to lower risk of several metabolic and vascular diseases. A recent meta-analysis^53^ of 14 RCTs showed reduced weight and body fat associated with whey protein supplementation.

This meta-analysis presents several strengths. The broad search of relevant studies, as was mentioned above, minimized the potential impacts of publication bias, the inclusion of a large number of studies enhanced the statistical power, the comprehensive analyses according to multiple study characteristics lead to more reliable results, and the identification of the current studies from different global regions supported generalizability of our findings.

However, several limitations should also be considered when interpreting the results of this meta-analysis. First, confounding factors that could be inherent in the primary studies may have biased our findings. In particular, dairy consumption often clusters with a better overall dietary profile. However, in the stratification by adjustment for important lifestyle and dietary factors including smoking, alcohol consumption, physical activity and dietary intakes, the inverse association between dairy consumption and risk of MetS remained. Second, a large proportion of studies used self-reported diet information, which may have introduced measurement error, resulting in misclassification of exposure. Given that most participants were not aware of their disease status (especially in prospective studies) before reporting dietary intakes, this misclassification would likely to be non-differential and lead to an underestimation of the magnitude of association. This may in part explain the results of subgroup analysis showing that studies assessing dietary intakes by self-administered FFQ reported a weaker association than those by interview and food diary. Third, the classifications of dairy products were inconsistent across studies, and the types of dairy consumed also differed according to different populations. Furthermore, the benefits of dairy on MetS may vary by specific types, or by fat content (Supplementary table S3), but no additional meta-analyses on individual dairy except for milk could be performed given the limited data available. Thus, future prospective studies of specific dairy consumption and MetS risk are required.

Our findings are important for public health. Globally, a large proportion of population did not meet the recommendation for dairy consumption, particularly in some developing countries in Asia and Africa^54^. For example, Chinese National Nutrition and Health Survey (2002) data showed that daily dairy consumption was 65.8 g/day in china’s urban population, and 11.4 g/d in rural population, respectively^55^. Despite the increase in consumption with economic development in the past decade, the daily consumption of dairy products is still much lower than the amount recommended^55^. Except for the well-known benefits of dairy in bone health, our novel findings of an inverse dose-response association between dairy consumption and MetS risk, in combination with recent evidence on T2DM, CVD, and specific cancers, provide further supports for public health recommendations to increase dairy consumption to prevent series of chronic diseases.

In conclusion, findings from this meta-analysis support the beneficial effect of increasing dairy consumption on the prevention of MetS. Future prospective studies investigating specific type of dairy products, with good control for potential confounders, followed by well-designed RCTs examining the effects of dairy consumption on MetS incidence are required for definite conclusions.

## Methods

### Literature search

A literature search was performed on PubMed (January 1, 1966 to December 23, 2014) and EMBASE (January 1, 1980 to December 31, 2014) databases using the search strategy reported in Table 2, with core search involving foods intake, dietary factors, dairy and its subtypes, and MetS, along with specific terms for study design. The bibliographies of retrieved articles and previous reviews were also carefully hand searched for additional studies. The search had no restrictions on language or type (full reports, conference abstracts, or letters to editors) of publications. We did not contact relevant authors for additional information.

### Study selection

Studies that met the following criteria were considered: *(a)* the study design was cohort, case-cohort, nested case-control, case-control or cross-sectional; *(b)* the exposure of interest was dairy products (including individual dairy products); *(c)* the outcome of interest was MetS; *(d)* recruiting adult population (aged ≥ 18 years); and *(e)* odds ratio (ORs) or relative risks (RRs) with corresponding 95% confidence interval (CIs) were reported or could be estimated. Studies that reported dairy consumption as a continuous variable were excluded because the risk estimates were not comparable with studies that used categorized or per unit increased dairy measures. For two publications^28^,^56^ from the same cohort, the one^28^ with longer duration was included in the primary analyses. The other was included in the subgroup analysis because the results for milk consumption were not reported in the one with longer duration. We did not exclude Cross-sectional studies in which the same populations were used in cohort studies because studies of different designs were separately analyzed.

### Data extraction

Using a standardized data-collection form, the following data were extracted from each included study: the first author’s last name, publication year, country of origin, dataset, sample size, age and sex of participants, type of dairy, levels of exposure, the maximally adjusted risk estimates with corresponding 95% CI for each category of exposure, methods for exposure ascertainment and outcome assessment, and variables accounted for in the statistical model. Literature search, study selection and data extraction were conducted independently by two authors (G-CC and L-QQ), with any disagreement resolved by consensus. Instead of using a quality score to assess methodological quality, we conducted various subgroup analysis to investigate whether the overall findings were significantly influenced by the specific study characteristics that are indicators of study quality (e.g, methods for exposure ascertainment and outcome assessment, and potential confounding factors adjusted for).

### Statistical analysis

The common measure of association was OR in cross-sectional and case-control studies, and RR in cohort studies. A random-effects model, which considers both within-and between-study variation was assigned to calculate the summary risk estimates. Heterogeneity test was performed using Q and *I*^2^ statistics. For the Q statistic, *P < *0.1 was considered as statistically significant; and for the *I*^2^ statistic, the following conventional cut-off points were used: <5% (low heterogeneity), 25–50% (moderate heterogeneity) and >75% (severe heterogeneity). Potential publication bias was investigated with the Egger’s test^57^.

Any results separately reported by population characteristics (e.g., sex or by body mass index) were pooled with the fixed-effect model before including in the meta-analysis. For the US study by Beydoun *et al.*^13^ that reported the OR as per unit increase in dairy consumption, a new OR with 95% CI were calculated for a four-serving increment, which were the means of the upper dairy categories of other studies from the USA. For the study by Fumeron *et al.*^30^ that used two criteria (the International Diabetes Federation [IDF] and the Adult Treatment Panel III of the National Cholesterol Education Program [NCEP ATP-III]) to diagnose MetS, the results according to NCEP ATP-III were used to be consistent with most studies included. To explore potential sources of heterogeneity, subgroup and meta-regression analyses were performed according to various study and population characteristics including: geographic region, sex, duration of follow-up (for cohort studies), methods for exposure ascertainment and outcome assessment, subtypes of dairy products, and adjustments for potential confounders. To examine the impacts of individual studies on the overall results, sensitivity analyses were carried out by omitting one study at each turn while pooling the results from the remainder. Further analyses were conducted by restricting to the studies that were published in English and to those published as full reports. A further attempt was made to combine all studies included (excluding two overlapping cross-sectional studies^19^,^24^).

To capture the detailed nature of the association, a dose-response analysis was also conducted by use of the method proposed by Greenland and Longnecker^58^ and Orsini *et al.*^59^. This method requires the number of cases and controls (or person-years in cohort studies) and the risk estimates with their variance estimates for at least three quantitative exposure categories. For the studies that did not provide the number of cases and controls (or person-years) in each exposure category, the data were estimated from total number of cases and controls (or person-years), e.g, the total number of person-years was divided by four if the data is analyzed by quartiles or five if the data are analyzed by quintiles. For each study, the median or mean level of consumption for each category was assigned to each corresponding risk estimate. When the median or mean consumption per category was not provided, the midpoint of the upper and lower boundaries in each category was assigned as average consumption. If the highest or lowest category was open-ended, the width of the interval was assumed to be the same as in the closest category. Most included studies reported dairy in servings, and for the studies^26^,^29^ that reported results in grams (g) or milliliters (ml) per day, the consumptions were converted into servings by using 200 g or ml as a serving size. A potential nonlinear relationship between dairy consumption and the development of MetS was examined by modeling exposure levels using restricted cubic splines with three knots at percentiles 10%, 50% and 95% of the distribution^60^. The *P*-value for nonlinearity was calculated by testing the null hypothesis that the coefficient of the second spline is equal to zero. All statistical analyses were performed using STATA software, version 11.0 (STATA Corp., College Station, TX, USA). All *P*-values were two-sided, and the level of significance was at <0.05, unless explicitly stated.

## Additional Information

**How to cite this article**: Chen, G.-C. *et al.* Dairy products consumption and metabolic syndrome in adults: systematic review and meta-analysis of observational studies. *Sci. Rep.* **5**, 14606; doi: 10.1038/srep14606 (2015).

## Supplementary Material

Supplementary Information

## Figures and Tables

**Table 1:** Results of subgroup analysis stratified by study and population characteristics and type of dairy.

		Cross-sectional/case-control studies (*N* = 16)					Prospective cohort studies (*N* = 7)				
		*N*	OR (95% CI)	*P*^a^	*I*^2^(%)	*P*^b^	*N*	RR (95% CI)	*P*^a^	*I*^2^(%)	*P*^b^
Region											
Asia-pacific		6	0.83 (0.76–0.91)	0.77	0.0		3	0.80 (0.66–0.95)	0.85	0.0	
Europe		5	0.81 (0.53–1.25)	0.003	75.6	0.83^c^	2	0.89 (0.79–1.00)	0.90	0.0	0.39^c^
USA		5	0.83 (0.60–1.15)	0.005	73.2	0.89^c^	2	0.75 (0.51–1.12)	0.10	63.9	0.98^c^
Sex											
Men		6	0.70 (0.48–1.04)	0.000	77.5		0	NA	NA	NA	
Women		5	0.92 (0.59–1.44)	0.009	70.3	0.41^d^	0	NA	NA	NA	NA
Both		10	0.86 (0.76–0.97)	0.16	31.0	0.63^d^	7	0.86 (0.79–0.92)	0.65	0.0	NA
Duration of follow-up											
≥9 years		NA	NA	NA	NA	NA	4	0.86 (0.78–0.95)	0.31	15.7	0.52
<9 years		NA	NA	NA	NA	NA	3	0.81 (0.68–0.96)	0.94	0.0	
Dairy ascertainment											
Self-report		8	0.85 (0.64–1.14)	0.000		0.70	4	0.88 (0.79–0.99)	0.91	0.0	0.49
Interview or food diary		7	0.82 (0.75–0.91)	0.69	0.0		3	0.82 (0.71–0.94)	0.22	34.3	
MetS assessment											
NCEP ATP-III		10	0.85 (0.75–0.95)	0.04	48.0	0.56	4	0.86 (0.78–0.95)	0.82	0.0	0.77
Other		5	0.65 (0.34–1.23)	0.002	76.7		3	0.76 (0.57–1.02)	0.20	37.1	
Type of dairy											
Total dairy		12	0.81 (0.70–0.94)	0.03	49.4	0.45	6	0.86 (0.79–0.92)	0.65	0.0	0.20
Milk		6	0.90 (0.74–1.11)	0.003	71.9		3	0.75 (0.63–0.89)	0.92	0.0	
Adjustment											
Smoking	*Yes*	12	0.80 (0.69–0.93)	0.01	55.0	0.46	7	0.86 (0.79–0.92)	0.65	0.0	NA
	*No*	4	0.93 (0.63–1.37)	0.02	69.2		0	NA	NA	NA	
Alcohol	*Yes*	6	0.77 (0.67–0.88)	0.21	30.5	0.36	4	0.78 (0.66–0.92)	0.59	0.0	0.25
	*No*	10	0.88 (0.72–1.08)	0.004	63.3		3	0.88 (0.81–0.95)	0.75	0.0	
Physical activity	*Yes*	11	0.86 (0.75–0.99)	0.003	62.4	0.32	7	0.86 (0.79–0.92)	0.65	0.0	NA
	*No*	5	0.70 (0.51–0.96)	0.15	50.4		0	NA	NA	NA	
Education	*Yes*	6	0.90 (0.73–1.10)	0.18	33.8	0.54	4	0.83 (0.75–0.92)	0.38	2.5	0.50
	*No*	10	0.80 (0.68–0.94)	0.003	63.9		3	0.88 (0.79–0.99)	0.76	0.0	
Dietary energy	*Yes*	13	0.78 (0.67–0.91)	0.02	50.2	0.22	6	0.83 (0.76–0.92)	0.63	0.0	0.45
	*No*	3	0.99 (0.74–1.32)	0.01	77.5		1	0.89 (0.79–1.00)	NA	NA	
Dietary fat	*Yes*	2	0.73 (0.59–0.90)	0.15	52.4	0.42	3	0.80 (0.59–1.07)	0.21	35.4	0.75
	*No*	14	0.85 (0.74–0.99)	0.007	54.9		4	0.85 (0.77–0.93)	0.81	0.0	
Meat	*Yes*	2	0.80 (0.70–0.93)	0.44	0.0	0.76	3	0.82 (0.71–0.94)	0.22	34.3	0.49
	*No*	14	0.83 (0.72–0.97)	0.001	62.1		4	0.88 (0.79–0.99)	0.91	0.0	
Whole/refined grain	*Yes*	1	0.81 (0.73–0.93)	NA	NA	0.94	3	0.82 (0.71–0.94)	0.22	34.3	0.49
	*No*	15	0.83 (0.71–0.96)	0.001	60.0		4	0.88 (0.79–0.99)	0.91	0.0	
Dietary fiber	*Yes*	3	0.88 (0.74–1.05)	0.40	0.0	0.78	2	0.58 (0.38–0.89)	0.88	0.0	0.13
	*No*	13	0.82 (0.70–0.95)	0.001	63.1		5	0.87 (0.80–0.93)	0.92	0.0	
Fruit and vegetable	*Yes*	2	0.80 (0.70–0.93)	0.44	0.0	0.76	3	0.82 (0.71–0.94)	0.22	0.0	0.49
	*No*	14	0.83 (0.72–0.97)	0.001	62.1		4	0.88 (0.79–0.99)	0.91	0.0	

CI, confidence interval; NA, not applicable; NCEP ATP-III, Adult Treatment Panel III of the National Cholesterol Education Program; OR, odds ratio; RR, relative risk.

^a^*P* value for heterogeneity among studies.

^b^*P* value for heterogeneity between groups according to meta-regression.

^c^Asia-pacific studies as a reference group.

^d^Male data as reference group.

**Table 2:** Literature search strategy for meta-analysis.

**#1:** food OR food groups OR diet OR dietary intake OR dietary consumption OR dietary factors;
**#2:** dairy OR milk OR cheese OR butter OR cream;
**#3**: insulin resistance syndrome OR metabolic syndrome OR syndrome X;
**#4:** cohort OR prospective OR case-cohort OR case-control OR cross-sectional OR retrospective;
**#5:** (#1 OR #2) AND #3 AND #4.
