# Supplementary material for: Dairy products consumption and metabolic syndrome in adults: systematic review and meta-analysis of observational studies
Source: Sci Rep. 2015 Sep 29;5:14606. doi: 10.1038/srep14606 (PMC4586521; doi:10.1038/srep14606)
Supplement: Supplementary Information [file srep14606-s1.pdf]

Dairy products consumption and metabolic syndrome in adults: systematic review and meta-analysis of observational studies

Guo-Chong Chen[1], Ignatius M.Y. Szeto [2], Li-Hua Chen [1], Shu-Fen Han [1], Yan-Jie Li [2], Rina van Hekezen[3], Li-Qiang Qin[1]

[1]Department of Nutrition and Food Hygiene, School of Public Health, Soochow University, Suzhou, 215123, China

[2]Yili Innovation Center, Inner Mongolia Yili Industrial Group Co., Ltd., Hohhot, 010110,. China

[3]Yili R&D Center (The Netherlands) B.V., 6708 PB Wageningen, Netherlands.

Supplementary figure captions:

Supplementary Figure S1. Sensitivity analysis for cross-sectional/case-control studies, omitting one at a time with the remaining studies pooled.

Supplementary Figure S2. Sensitivity analysis for prospective cohort studies, omitting one at a time with the remaining studies pooled.

Supplementary Table S1. Characteristics of excluded studies and reasons for exclusion

Study	Design	Results for diary	Reasons for exclusion
van Meijl, 2009[1]	Cross-sectional	Low-fat dairy is beneficial for systolic blood pressure, but not other MetS parameters.	Investigating individual parameters of MetS.
Samara, 2012[2]	Cohort	Dairy improve metabolic profile in men but not in women.	Investigating individual parameters of MetS.
te Velde, 2011[3]	Cohort	Diary intake during age 13 to 36 years did not affect MetS risk at age 36 years.	Including adolescent population.
Shin, 2009[4]	Cohort	Dairy and milk intake inversely associated with MetS. Dairy: adjusted RR=0.75 (95% CI=0.64-0.88); Milk: adjusted RR=0.79 (95% CI=0.67-0.92)	Duplicate publication (results for milk was included in the meta-analysis).
Yoo, 2004[5]	Cross-sectional	Low-fat dairy intake was higher in subjects who had no risk factors for MetS than those who had 1 to 2 risk factors.	Investigating individual parameters of MetS.
Høstmark, 2012[6]	Cross-sectional	Cheese intake inversely associated with MS, adjusted OR=0.97 (95% CI=0.94-0.99)	Dairy analyzed as a continuous variable.
Uenishi, 2010[7] (in Japanese)	Cross-sectional	Calcium intake from dairy reduce MetS in non-smoking women (adjusted OR=0.60, 95% CI=0.41-0.87), but not in smoking men (adjusted OR=1.13, 95% CI=0.85- 1.51) or non-smoking men (adjusted OR=0.80, 95% CI=0.60 -1.06)	Using calcium as an indicator of dairy intake.
Kouki, 2011[8]	Cross-sectional	None.	No results on dairy.
Høstmark, 2011[9]	Cross-sectional	Cheese intake inversely associated with MS, adjusted OR=0.96 (95% CI=0.95-0.97)	Dairy analyzed as a continuous variable
Wang , 2011[10] (in Chinese)	Case-control	Dairy intake inversely associated with MS, adjusted OR=0.78 (95% CI=0.62-0.99)	Dairy analyzed as a continuous variable
Al-Daghri, 2013[11]	Cross-sectional	None.	No results on dairy.
Bain, 2013[12]	Case-control	None.	No results on dairy.
Bruscato, 2010[13]	Cross-sectional	None.	No results on dairy.
Carnethon, 2004[14]	Cohort	None.	No results on dairy.
Eilat-Adar, 2008[15]	Cross-sectional	None.	No results on dairy.
Cabello-Saavedra, 2010[16]	Cross-sectional	None.	No results on dairy.
Kwaśniewska, 2009[17]	Cross-sectional	None.	No results on dairy.
Michael, 2008[18]	Cross-sectional	None.	No results on dairy.
Mirmiran, 2008[19]	Cohort	None.	No results on dairy.
Motamed, 2013[20]	Cross-sectional	None.	No results on dairy.
Prasad, 2012[21]	Cross-sectional	None.	No results on dairy.
Reppert, 2008[22]	Cross-sectional	None.	No results on dairy.

CI, confidence interval; MetS, metabolic syndrome; OR, odds ration; RR, relative risk.

References

1. van Meijl, L.E. & Mensink, R.P. Low-fat dairy consumption reduces systolic blood pressure, but does not improve other metabolic risk parameters in overweight and obese subjects. *Nutr Metab Cardiovasc Dis* **21**, 355-361 (2011).
2. Samara, A. *et al.* Dairy product consumption, calcium intakes, and metabolic syndrome-related factors over 5 years in the STANISLAS study. *Nutrition* **29**, 519-524 (2013).
3. te Velde, S.J. *et al.* Dairy intake from adolescence into adulthood is not associated with being overweight and metabolic syndrome in adulthood: the Amsterdam Growth and Health Longitudinal Study. *J Hum Nutr Diet* **24**, 233-244 (2011).
4. Shin, H., Yoon, Y.S., Lee, Y., Kim, C.I. & Oh, S.W. Dairy product intake is inversely associated with metabolic syndrome in Korean adults: Anseong and Ansan cohort of the Korean Genome and Epidemiology Study. *J Korean Med Sci* **28**, 1482-1488 (2013).
5. Yoo, S. *et al.* Comparison of dietary intakes associated with metabolic syndrome risk factors in young adults: the Bogalusa Heart Study. *Am J Clin Nutr* **80**, 841-848 (2004).
6. Hostmark, A.T. & Haug, A. Does cheese intake blunt the association between soft drink intake and risk of the metabolic syndrome? Results from the cross-sectional Oslo Health Study. *BMJ Open* **2**(2012).
7. Uenishi, K. *et al.* Milk, Dairy Products and Metabolic Syndrome: A Cross-sectional Study of Japanese. *J Jpn Soc Nutr Food Sci* **63**, 151-159 (2010).
8. Kouki, R. *et al.* Food consumption, nutrient intake and the risk of having metabolic syndrome: the DR's EXTRA Study. *Eur J Clin Nutr* **65**, 368-377 (2011).
9. Hostmark, A.T. & Tomten, S.E. The Oslo health study: cheese intake was negatively associated with the metabolic syndrome. *J Am Coll Nutr* **30**, 182-190 (2011).
10. Wang, Y.S. *et al.* [A matched case-control study on the risk factors of metabolic syndrome among policemen]. *Zhonghua Lao Dong Wei Sheng Zhi Ye Bing Za Zhi* **29**, 567-571 (2011).
11. Al-Daghri, N.M. *et al.* Selected dietary nutrients and the prevalence of metabolic syndrome in adult males and females in Saudi Arabia: a pilot study. *Nutrients* **5**, 4587-4604 (2013).
12. Bian, S. *et al.* Dietary nutrient intake and metabolic syndrome risk in Chinese adults: a case-control study. *Nutr J* **12**, 106 (2013).
13. Bruscato, N.M. *et al.* Dietary intake is not associated to the metabolic syndrome in elderly women. *N Am J Med Sci* **2**, 182-188 (2010).
14. Carnethon, M.R. *et al.* Risk factors for the metabolic syndrome: the Coronary Artery Risk Development in Young Adults (CARDIA) study, 1985-2001. *Diabetes Care* **27**, 2707-2715 (2004).
15. Eilat-Adar, S. *et al.* Sex may modify the effects of macronutrient intake on metabolic syndrome and insulin resistance in American Indians: the strong heart study. *J Am Diet Assoc* **108**, 794-802 (2008).
16. Cabello-Saavedra, E. *et al.* Macronutrient intake and metabolic syndrome in subjects at high cardiovascular risk. *Ann Nutr Metab* **56**, 152-159 (2010).
17. Kwasniewska, M., Kaleta, D., Dziankowska-Zaborszczyk, E. & Drygas, W. Healthy behaviours, lifestyle patterns and sociodemographic determinants of the metabolic syndrome. *Cent Eur J Public Health* **17**, 14-19 (2009).
18. Skilton, M.R., Laville, M., Cust, A.E., Moulin, P. & Bonnet, F. The association between dietary macronutrient intake and the prevalence of the metabolic syndrome. *Br J Nutr* **100**, 400-407 (2008).
19. Mirmiran, P., Noori, N. & Azizi, F. A prospective study of determinants of the metabolic syndrome in adults. *Nutr Metab Cardiovasc Dis* **18**, 567-573 (2008).
20. Motamed, S. *et al.* Micronutrient intake and the presence of the metabolic syndrome. *N Am J Med Sci* **5**, 377-385 (2013).
21. Prasad, D.S., Kabir, Z., Dash, A.K. & Das, B.C. Prevalence and risk factors for metabolic syndrome in Asian Indians: A community study from urban Eastern India. *J Cardiovasc Dis Res* **3**, 204-211 (2012).
22. Reppert, A., Steiner, B.F. & Chapman-Novakofski, K. Prevalence of metabolic syndrome and associated risk factors in Illinois. *Am J Health Promot* **23**, 130-138 (2008).

Supplementary Table S2. Characteristics of included cross-sectional (*N*=15) and case-control (*N*=1) studies on dairy products consumption and MetS.

Study	Dataset (Country)	Participants	Type of dairy	Comparison	OR (95% CI)	Dairy assessment	MetS assessment	Variables accounted for
Mennen, 2000[1]	DESIR (France)	4976 M/F aged 30-64 yr	Dairy	>4 vs.≤1portions/d	M: 0.63 (0.40-0.99) F: 0.76 (0.47-2.66)	Self-administered FFQ	≥2 of the 4 components: high fasting glucose, high triglycerides, high BP, low HDL cholesterol.	Age, WHP, smoking, and intakes of energy and alcohol.
Lawlor, 2005[2]	BWHHS (UK)	4024 F aged 60-79yr	Milk	Drinker vs. non-drinker	1.82 (1.06-3.03)	Self-administered FFQ	Modified WHO definition	Age, physical activity, smoking, dietary characteristics, and 10 indicators of socioeconomic position.
Azadbakht, 2005[3]	TLGS (Iran)	827 M/F aged 18-74 yr	Dairy	≥3.1 vs.<1.7 ser/d	0.82 (0.63-0.99)	Interview-based FFQ	NCEP ATP-Ⅲ	Age, BMI, physical activity, smoking, use of BP and oestrogen medication, and intakes of energy, fat, calcium and protein.
Liu, 2005[4]	WHS (USA)	10066 F aged≥ 45yr	Dairy	>3.0 vs.<0.91 ser/d	0.66 (0.55-0.80)	Self-administered FFQ	Modified NCEP ATP-Ⅲ	Age, smoking, physical activity, multivitamin use, parental history of MI, glycemic load, and intakes of total energy, alcohol, total fat, cholesterol and protein.
Elwood, 2007[5]	Caerphilly cohort (UK)	2375 M aged 45-59 yr	Milk	>1pint/d vs. little or none.	0.38 (0.18-0.78)	Self-administered FFQ	Modified WHO definition	Age, smoking, energy intake and social class.
Ruidavets, 2007[6]	French MONICA centers (France)	912 M aged 45-64yr	Dairy	Q5 vs. Q1 (median intake:175 g/d)	0.64 (0.37-1.09)	Food diary	NCEP ATP-Ⅲ	Age, smoking, physical activity, education, center, dieting, drugs for hypertension and dyslipidaemia, intakes of energy and alcohol, and diet quality index.
Snijder, 2007[7]	Hoorn Study (the Netherlands)	2064 M/F aged 50-75 yr	Dairy	≥5.57 vs.≤2.90 ser/d	1.01 (0.74-1.39)	Self-administered FFQ	NCEP ATP-Ⅲ	Age, sex, physical activity, smoking, education, income, antihypertensive medication use, and intakes of energy, alcohol and fiber.
Beydoun, 2008[8]	NHANES (USA)	4519 M/F aged ≥18yr	Dairy	Each 1 ser/d	1.05 (0.97-1.14)	Self-administered FFQ	NCEP ATP-Ⅲ	Age, sex, and physical activity, ethnicity, socioeconomic status, and energy intake,.
Shin, 2009[9]	National Cancer Center (Korea)	5337 M aged≥ 30yr	Dairy	>1ser/d vs.<2-3 ser/mo	0.92 (0.75-1.12)	Self-administered FFQ	Modified NCEP ATP-Ⅲ	Age, smoking, physical activity and family history of T2D.
Troy, 2010[10] (abstract)	Framingham Offspring Study (USA)	3104 M/F, age N.R	Dairy	>3.0 vs.<0.4 ser/d	0.78 (0.60-0.99)	N.R	N.R	Age, sex, smoking, and intakes of energy and alcohol.

Zhang, 2010[11] (in Chinese)	Communities in Guangzhou Municipal People's Street (China)	459 M/F aged 40-79 yr	Dairy	M: 128 vs. 0 ml/d; F: 219 vs. 0 ml/d	0.51 (0.16-1.68)	Interview-based FFQ	IDF	Age, sex, smoking, education, income, marital status, and intakes of energy, meat, egg, fruit, vegetable, and soy products.
Kwon, 2010[12]	KNHANES Ⅲ (Korea)	4890 M/F aged 47.1 yr	Milk	≥1 ser/d vs. rarely	0.85 (0.68-1.06)	Interview-based FFQ	Modified NCEP ATP-Ⅲ	Age, sex, BMI,smoking, physical activity, education, and intakes of energy, alcohol and fiber.
de Oliveira, 2012[13]	Move for Health program (Brazil)	305 M/F aged 54.2 (cases) or 55.8 (non-cases) yr	Dairy	≥3 vs.<3 ser/d	1.6 (0.64-4.03)	Self-administered FFQ	Modified NCEP ATP-Ⅲ	Age, sex, BMI, and energy intake.
Hosseinpour-Niazi, 2013[14] (in Persian, case-control)	Subjects consulting health problems in a hospital (Iran)	320 M/F aged 41.3 yr	Dairy	≥5.7 vs.≤ 3.1 ser/d	0.6 (0.2-0.9)	Interview-based FFQ	NCEP ATP-Ⅲ	Age, sex, BMI, smoking, physical activity, education, and intakes of energy, cholesterol and fiber.
Mosley, 2013[15]	2009 UP AMIGOS cohort (Mexico)	339 M/F aged 18-25 yr	Dairy	≥3 vs.<3 ser/d	0.34 (0.12-1.00)	Self-administered FFQ	IDF/AHA joint criteria	Age, sex, physical activity, family history of CVD and T2DM, and energy intake.
Sadeghi, 2014[16]	IHHP (Iran)	1752 M/F with a mean age of 37.8 yr (<7 ser/wk) or 39.1yr (≥7 ser/ wk)	Cheese	≥7 vs.<7 ser/wk	0.81 (0.71-0.94)	Interview-based FFQ	Modified NCEP ATP-Ⅲ	Age, sex, BMI, physical activity, dietary index, and intakes of oil, grain, pulses, fruit and vegetable, meat, and dairy.

AHA, American Heart Association; BMI, body mass index; BP, blood pressure; BWHHS, British Women's Heart and Health Study; CI, confidence interval; d, day; CVD, cardiovascular diseases; DESIR, Data from an Epidemiological Study on the Insulin Resistance syndrome; F, females; FFQ, food frequency questionnaire; IDF, International Diabetes Federation; mo, month; IHHP, Isfahan Health Heart Program; KNHANES, Korea National Health and Nutrition Examination Survey; M, males; MI, myocardial infarction; MetS, metabolic syndrome; NCEP ATP-III, Adult Treatment Panel III of the National Cholesterol Education Program; NHANES, National Health and Nutrition Examination Survey; N.R., not reported; OR, odds ratio; RR, relative risk; ser, servings; TLGS, Tehran Lipid and Glucose Study; T2DM, type 2 diabetes; UP AMIGOS, Universities of San Luis Potosí and Illinois: A Multidisciplinary Investigation on Genetics, Obesity, and Social-Environment; WHS, Women's Health Study; wk, week; WHO, World Health Organization; WHP, waist-hip ratio; yr, years.

References

1. Mennen, L.I. *et al.* Possible protective effect of bread and dairy products on the risk of the metabolic syndrome. *Nutr Res* **20**, 335-347 (2000).
2. Lawlor, D.A., Ebrahim, S., Timpson, N. & Davey Smith, G. Avoiding milk is associated with a reduced risk of insulin resistance and the metabolic syndrome: findings from the British Women's Heart and Health Study. *Diabet Med* **22**, 808-811 (2005).
3. Azadbakht, L., Mirmiran, P., Esmaillzadeh, A. & Azizi, F. Dairy consumption is inversely associated with the prevalence of the metabolic syndrome in Tehranian adults. *Am J Clin Nutr* **82**, 523-530 (2005).
4. Liu, S. *et al.* Dietary calcium, vitamin D, and the prevalence of metabolic syndrome in middle-aged and older U.S. women. *Diabetes Care* **28**, 2926-2932 (2005).
5. Elwood, P.C., Pickering, J.E. & Fehily, A.M. Milk and dairy consumption, diabetes and the metabolic syndrome: the Caerphilly prospective study. *J Epidemiol Community Health* **61**, 695-698 (2007).
6. Ruidavets, J.B. *et al.* High consumptions of grain, fish, dairy products and combinations of these are associated with a low prevalence of metabolic syndrome. *J Epidemiol Community Health* **61**, 810-817 (2007).
7. Snijder, M.B. *et al.* Is higher dairy consumption associated with lower body weight and fewer metabolic disturbances? The Hoorn Study. *Am J Clin Nutr* **85**, 989-995 (2007).
8. Beydoun, M.A. *et al.* Ethnic differences in dairy and related nutrient consumption among US adults and their association with obesity, central obesity, and the metabolic syndrome. *Am J Clin Nutr* **87**, 1914-1925 (2008).
9. Shin, A., Lim, S.Y., Sung, J., Shin, H.R. & Kim, J. Dietary intake, eating habits, and metabolic syndrome in Korean men. *J Am Diet Assoc* **109**, 633-640 (2009).
10. Troy, L.M., Jacques, P.F., Vasan, R.S. & McKeown, N.M. Dairy intake not associated with metabolic syndrome but milk and yogurt intake is inversely associated with prevalence of hypertension in middle-aged adults. *FASEB Journal* **24** (conference abstract) (2010).
11. Zhang, B. *et al.* Cross-sectional Study on Relationship between Dairy Consumption and Metabolic Syndrome. *J SUN Yat-sen Univ (Med Sci)* **31** 582-587 (in Chinese) (2010).
12. Kwon, H.T. *et al.* Milk intake and its association with metabolic syndrome in Korean: analysis of the third Korea National Health and Nutrition Examination Survey (KNHANES III). *J Korean Med Sci* **25**, 1473-1479 (2010).
13. de Oliveira, E.P., McLellan, K.C., Vaz de Arruda Silveira, L. & Burini, R.C. Dietary factors associated with metabolic syndrome in Brazilian adults. *Nutr J* **11**, 13 (2012).
14. Hosseinpour-Niazi, S., Mirmiran, P., Ejtahed, H., Nakhoda, K. & Azizi, F. Food groups, inflammatory markers and the metabolic syndrome in adults. *Iran J Endocrinol Metab* **15**, 340-351 (in Persian) (2013).
15. Mosley, M.A., Andrade, F.C., Aradillas-Garcia, C. & Teran-Garcia, M. Consumption of Dairy and Metabolic Syndrome Risk in a Convenient Sample of Mexican College Applicants. *Food and Nutrition Sciences* **4**, 56 (2013).
16. Sadeghi, M. *et al.* Cheese consumption in relation to cardiovascular risk factors among Iranian adults- IHHP Study. *Nutr Res Pract* **8**, 336-341 (2014).

Supplementary Table S3. Characteristics of included prospective cohort studies (*N*=7) on dairy products consumption and MetS.

Study	Dataset (Country)	Duration, yr	Participants	Type of dairy	Comparison	RR (95% CI)	Dairy assessment	MetS assessment	Variables accounted for
Pereira, 2002[1]	CARDIA (USA)	10	3157 M/F aged 18-30 yr	Dairy	≥35 vs. 0-10 ser/wk	BMI≥25: 0.38 (0.17-0.83) BMI<25: 0.72 (0.39-1.34)	Food diary	≥2 of the 4 components: abnormal glucose homeostasis, obesity, elevated BP, and dyslipid.	Age, sex, BMI, smoking, race, center, education, physical activity, vitamin supplement, and intakes of energy, alcohol, polyunsaturated fat, caffeine, dietary fiber, whole and refined grains, meat, fruit, vegetables, magnesium, calcium, and vitamin D.
Damiao, 2006[2]	JBDS (Japan)	7	151 M/F aged 40-79 yr	Milk	223.7 vs. 12.4 g/d	0.93 (0.28-3.00)	Self-administered FFQ	Modified NCEP ATP-Ⅲ	Age, sex, physical activity, smoking, education, and intakes of alcohol, energy, fat, and fried foods.
Lutsey, 2008[3]	ARIC (USA)	9	9514 M/F aged 45-64 yr	Dairy	3.30 vs. 0.28 ser/d	0.87 (0.77-0.98)	Interview-based FFQ	AHA guidelines	Age, sex, smoking, physical activity, race, education, center, and intakes of energy, meat, fruit, vegetables, whole grains, and refined grains.
Snijder, 2008[4]	Hoorn Study (the Netherland)	6.4	885 M/F aged 50-75 yr	Dairy	≥5.75 vs. ≤2.97 ser/d	0.86 (0.52-1.42)	Self-administered FFQ	NCEP ATP-Ⅲ	Age, sex, smoking, physical activity, and intakes of energy and alcohol.
Fumeron, 2011[5]	DESIR (France)	9	3435 M/Faged 30-65 yr	Dairy (except cheese)	>2 vs.<1 ser/d	IDF: 0.88 (0.79-0.97) NCEP ATP-Ⅲ: 0.89 (0.79-1.00)	Self-administered FFQ	IDF, NCEP ATP-Ⅲ	Age, sex, BMI, smoking, physical activity, and fat intake.
Louie, 2013[6]	BMES (Australia)	10	1824 M/F aged ≥49 yr	Dairy	3.1 vs. 0.5 ser/d	0.62 (0.24-1.62)	Self-administered FFQ	Modified IDF definition	Age, sex, Smoking, physical activity, dietary glycemic load, family history of T2D, and intakes of energy, fiber from vegetables, and calcium.
Baik, 2013[7]	KoGES (Korea)	6	5251 M/F aged 40-69 yr	Dairy	1.6 vs. 0 ser/d	0.80 (0.66-0.96)	Interview-based FFQ	Modified NCEP ATP-Ⅲ	Age, sex, smoking, physical activity, income, occupation, education, study sites, FTO genotypes, and intakes of alcohol, energy, refined grains and starches, mixed grain rice and cereal, fish and seafood, red meat and processed meat, poultry, eggs, legumes, nuts, fruit and vegetable, sweetened carbonated beverage, green tea, and coffee.

AHA, American Heart Association; ARIC, Atherosclerosis Risk in Communities; BMES, Blue Mountains Eye Study; BMI, body mass index; CARDIA, Coronary Artery Risk Development in Young Adults; CI, confidence interval; d, day; DESIR, Data from an Epidemiological Study on the Insulin Resistance syndrome; F, Females; FFQ, food frequency questionnaire; IDF, International Diabetes Federation; MetS, metabolic syndrome; JBDS, Japanese-Brazilian Diabetes Study; KoGES, Korean Genome Epidemiology Study; M, males; NCEP ATP, Adult Treatment Panel III of the National Cholesterol Education Program; RR, relative risk; ser, servings; yr, years.

References

1. Pereira, M.A. *et al.* Dairy consumption, obesity, and the insulin resistance syndrome in young adults: the CARDIA Study. *JAMA* **287**, 2081-2089 (2002).
2. Damiao, R., Castro, T.G., Cardoso, M.A., Gimeno, S.G. & Ferreira, S.R. Dietary intakes associated with metabolic syndrome in a cohort of Japanese ancestry. *Br J Nutr* **96**, 532-538 (2006).
3. Lutsey, P.L., Steffen, L.M. & Stevens, J. Dietary intake and the development of the metabolic syndrome: the Atherosclerosis Risk in Communities study. *Circulation* **117**, 754-761 (2008).
4. Snijder, M.B. *et al.* A prospective study of dairy consumption in relation to changes in metabolic risk factors: the Hoorn Study. *Obesity (Silver Spring)* **16**, 706-709 (2008).
5. Fumeron, F. *et al.* Dairy consumption and the incidence of hyperglycemia and the metabolic syndrome: results from a french prospective study, Data from the Epidemiological Study on the Insulin Resistance Syndrome (DESIR). *Diabetes Care* **34**, 813-817 (2011).
6. Louie, J.C. *et al*. Higher regular fat dairy consumption is associated with lower incidence of metabolic syndrome but not type 2 diabetes. *Nutr Metab Cardiovasc Dis* **23**, 816-821 (2013).
7. Baik, I., Lee, M., Jun, N.R., Lee, J.Y. & Shin, C. A healthy dietary pattern consisting of a variety of food choices is inversely associated with the development of metabolic syndrome. *Nutr Res Pract* **7**, 233-241 (2013).

Supplementary Table S4. Studies reporting results for the association between individual dairy products and MetS.

Study	Country	Reported results				
		Cheese	Yogurt	Butter	High-fat dairy	Low-fat dairy
Cross-sectional studies						
Liu, 2005[1]	USA	None	None	None	High-fat dairy (high vs. low): OR=0.71 (95% CI=0.58-0.87)	Low-fat dairy (high vs. low): OR=0.78 (95% CI=0.64-0.95)
Beydoun, 2008[2]	USA	Per 1 ser/d: OR=1.16 (95% CI=1.04-1.29)	Per 1 ser/d: OR=0.40 (95% CI=0.18-0.89)	None	Whole milk (per 100 g/d): OR=0.98 (95% CI=0.90-1.07)	Low fat milk (per 100 g/d): OR=1.02 (95% CI=0.97-1.06)
Mosley, 2013[3]	Mexico	High vs low: OR=0.59 (95% CI=0.29-1.43)	None	None	Whole milk (high vs. low): OR=0.63 (95%CI=0.29-1.25)	None
Sadeghi, 2014[4]	Iran	High vs low: OR=0.81 (95% CI=0.71-0.94)	None	None	None	None
Cohort studies						
Pereira, 2002[5]	USA	None	Per 1 ser/d: RR=0.58 (95% CI=0.20-1.66)	Per 1 ser/d: RR=0.90 (95% CI=0.76-1.05)	None	None
Fumeron, 2011[6]	France	High vs low: RR=0.82 (95% CI=0.71-0.95)	None	None	None	None
Louie, 2013[7]	Australia	None	None	None	Regular fat dairy (high vs. low): RR=0.39 (95% CI=0.22-0.70)	Regular/low fat dairy (high vs. low): RR=2.01 (95% CI=1.05-3.83)

CI, confidence interval; MetS, metabolic syndrome; OR, odds ration; RR, relative risk.

References

1. Liu, S. *et al*. Dietary calcium, vitamin D, and the prevalence of metabolic syndrome in middle-aged and older U.S. women. *Diabetes Care* **28**, 2926-2932 (2005).
2. Beydoun, M.A. *et al.* Ethnic differences in dairy and related nutrient consumption among US adults and their association with obesity, central obesity, and the metabolic syndrome. *Am J Clin Nutr* **87**, 1914-1925 (2008).
3. Mosley, M.A., Andrade, F.C., Aradillas-Garcia, C. & Teran-Garcia, M. Consumption of Dairy and Metabolic Syndrome Risk in a Convenient Sample of Mexican College Applicants. *Food and Nutrition Sciences* **4**, 56 (2013).
4. Sadeghi, M. *et al.* Cheese consumption in relation to cardiovascular risk factors among Iranian adults- IHHP Study. *Nutr Res Pract* **8**, 336-341 (2014).
5. Pereira, M.A. *et al.* Dairy consumption, obesity, and the insulin resistance syndrome in young adults: the CARDIA Study. *JAMA* **287**, 2081-2089 (2002).
6. Fumeron, F. *et al.* Dairy consumption and the incidence of hyperglycemia and the metabolic syndrome: results from a french prospective study, Data from the Epidemiological Study on the Insulin Resistance Syndrome (DESIR). *Diabetes Care* **34**, 813-817 (2011).
7. Louie, J.C. *et al.* Higher regular fat dairy consumption is associated with lower incidence of metabolic syndrome but not type 2 diabetes. *Nutr Metab Cardiovasc Dis* **23**, 816-821 (2013).

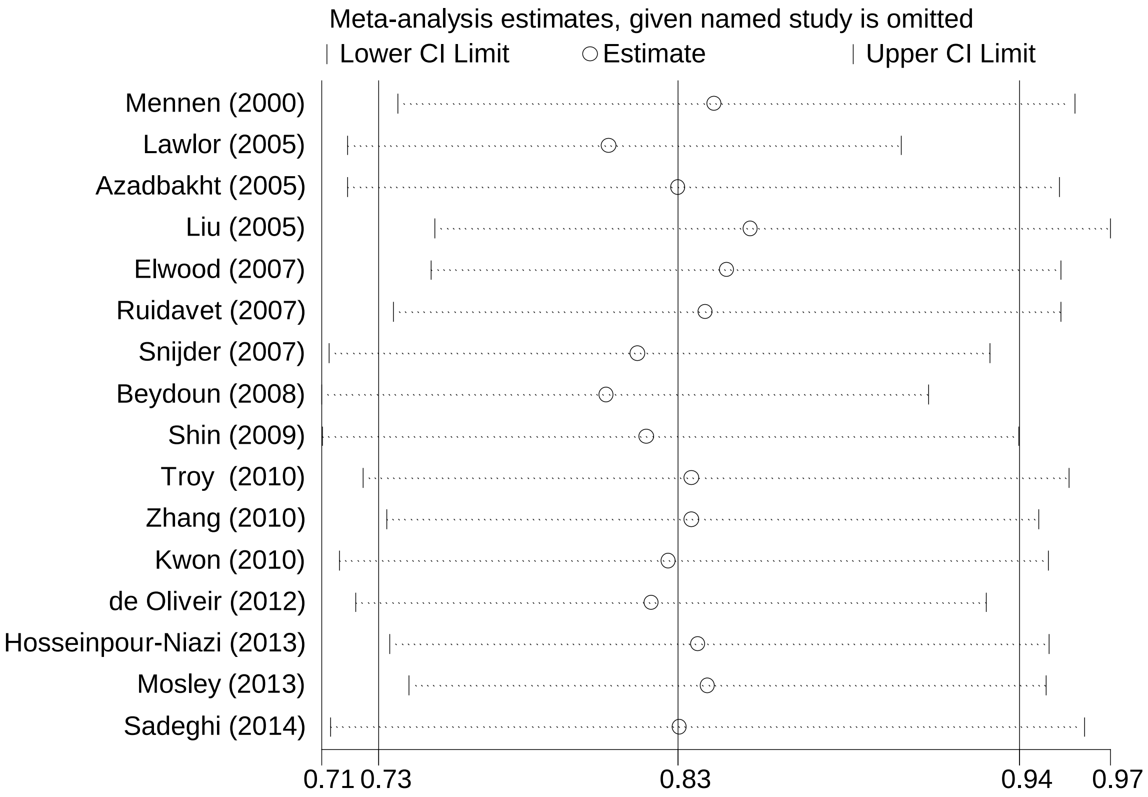

Supplementary Figure S1. Sensitivity analysis for cross-sectional/case-control studies, omitting one at a time with the remaining studies pooled.

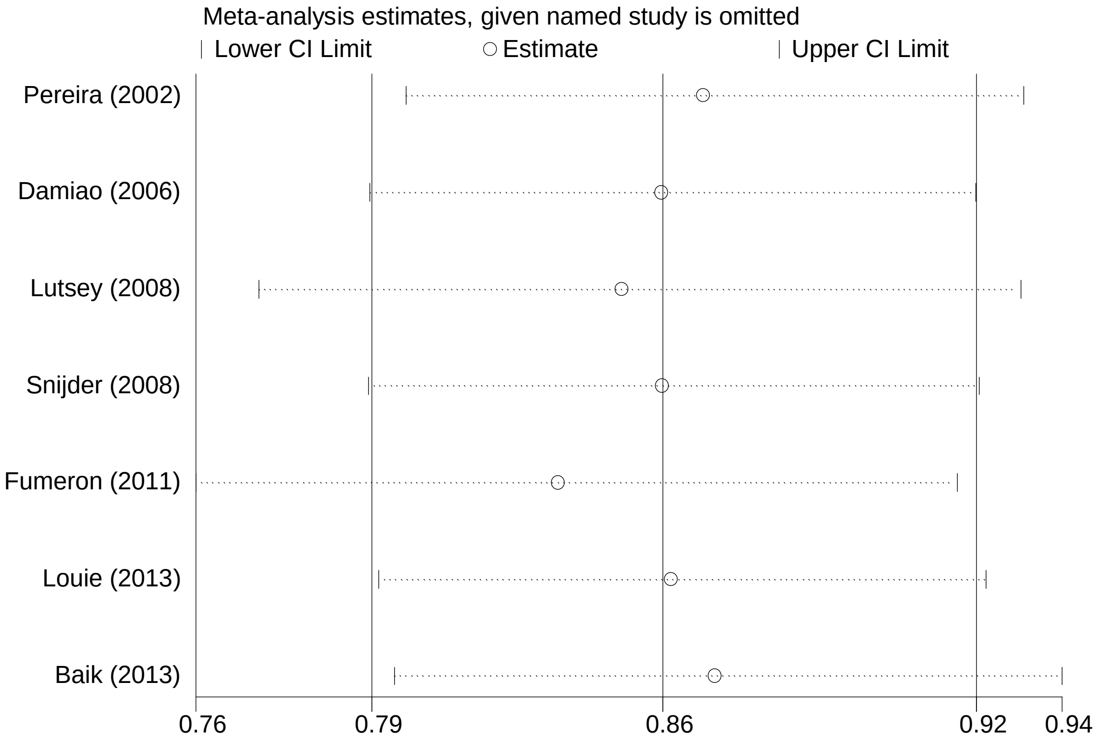

Supplementary Figure S2. Sensitivity analysis for prospective cohort studies, omitting one at a time with the remaining studies pooled.
